# Longitudinal association between sleep features and refractive errors in preschoolers from the EDEN birth-cohort

**DOI:** 10.1038/s41598-021-88756-w

**Published:** 2021-04-27

**Authors:** Alexis Rayapoullé, Claude Gronfier, Anne Forhan, Barbara Heude, Marie-Aline Charles, Sabine Plancoulaine

**Affiliations:** 1 CRESS, Inserm, INRAE, Université de Paris, 75004 Paris, France; 2 Hôpitaux de Paris, 3 rue Victoria, 75004 Paris, France; 3 Lyon Neuroscience Research Center (CRNL), Waking Team, Inserm UMRS 1028, CNRS UMR 5292, Université Claude Bernard Lyon 1, Université de Lyon, 69000 Lyon, France

**Keywords:** Risk factors, Medical research, Epidemiology, Paediatric research

## Abstract

Refractive errors are common, especially in children and adolescents, leading to global health issues, academic implications and economic costs. Circadian rhythm and sleep habits may play a role. The study included 1130 children from the EDEN birth-cohort. Data were collected through parental questionnaires at age 2 and 5 for sleep duration and timing, and at age 5 for refractive error. At 5 years, 20.4% were prescribed glasses (2% for myopia, 11.9% for hyperopia and 6.8% for unknown reason). Children slept on average (SD) 11h05/night (± 30 min) and 10h49/night (± 48 min) at age 2 and 5, respectively. Average bedtime and midsleep was 8.36 pm (± 30 min), 2.06 am (± 36 min), and 8.54 pm (± 30 min), 2.06 am (± 24 min) at age 2 and 5, respectively. A U-shaped association was observed between sleep duration at age 2 and eyeglass prescription at age 5. Later midsleep and bedtime at age 2 were associated with an increased risk of eyeglass prescription at age 5. Associations became borderline significant after adjustment for confounding factors. Sleep duration and timing at age 2 were associated with subsequent refractive errors in preschoolers from general population. Sleep hygiene might be a target for refractive errors prevention.

## Introduction

Emmetropization is an active mechanism that is still imperfectly understood by the scientific community. It is the result of a precise and active mechanism in the eyeball which combines the right axial length with the right corneal curvature, in order for distant images to be formed on the retina, with accommodation fully relaxed. For the past three decades, the scientists have studied the determinants of eye growth and shape in order to explain the origins of refractive errors^1–3^. Myopia, also known as nearsightedness, appears when the ocular axial length is too long for the optical power of the cornea and lens. On the contrary, hyperopia (or farsightedness) occurs when the axial length is too short and or the cornea too flat.

Myopia is, on the long run, significantly associated to ocular morbidity, especially cataract and glaucoma^4^. It is estimated that 25% of world population is currently myopic, and that this number could increase to 50% by 2050, making it a rising major public health concern^5^. Hyperopia may be a precursor of visual motor and sensory sequelae such as accommodative esotropia, anisometropia and unilateral or bilateral amblyopia^6^.

There is a lot of evidence suggesting that the emmetropization process reacts to visual stimuli. Regarding myopia, the increase of near-work activities appears to be a contributing factor^7–9^ while the exposure to outdoor natural light seems on the contrary to be protective^10,11^. However, associations with artificial light are conflicting. Studies on chicks seem relatively homogenous: rearing them under constant light tends to turn them hyperopic^12,13^, while rearing them under constant darkness turns them myopic at first but hyperopic on the long-term due to the excessive flattening of the cornea^14,15^. Studies on primates are a bit more contradictory: Troilo et al. found that sutured eyelid caused the eye underneath to become hyperopic at first, before ultimately growing to become myopic^16^. Smith et al. did not find an association between constant lighting and myopia on primates^17^. To extend these results, number of studies on humans have analyzed the association between night-light exposure during the first 2 years of life and the subsequent onset of myopia during childhood or adolescence, but get mixed results. Although two studies found positive associations^18,19^, six reported non-significant ones^9,20–24^.

It is also known that the human eye possesses intrinsic circadian rhythms^25–27^ in which dopamine and melatonin regulate—among other things—axial length and cones/rods coupling as shown in animal studies^28–30^. In addition to this, it was shown on baby monkeys that axial eye length increases during the day and reduces during the night (but growing overall in the long-term) and that this circadian rhythm was inverted during adolescence^31^. Ocular and systemic diurnal rhythms were also found to be robust in human children and related to axial eye length and choroidal thickness^32^. Studies in animals suggested that ocular diurnal rhythms (including axial length and choiroidal thickness) may have important implication in the eye growth regulation and in the development of refractive errors, especially myopia, when they are desynchronized^33,34^. Finally, one recent meta-analysis of 542,934 subjects of European ancestry identified some genetic factors controlling circadian rhythm and pigmentation for being responsible for refractive errors and myopia^35^.

The sleep–wake cycle is probably the most prominent feature of circadian rhythmicity. A disturbance in sleep may be the cause as well as the consequence of desynchronized circadian timing system. Additionally, inappropriate exposure to light over the 24 h is now recognized as a factor of sleep and circadian disturbance^36^. Four studies have investigated the association between sleep quality and myopia. Ayaki et al. found in a cross-sectional study on 278 children between 10 and 19 years old that Pittsburgh Sleep Quality Index (PQSI) score, an indicator for poor sleep quality, was positively correlated to myopia intensity^37^. Jee et al. found an inverse association between myopia and sleep duration in a cross-sectional study among 3625 12–19 year-old adolescents^38^. Similar results were shown on a larger cross-sectional study among 15,000 children from 7 to 18 years old^7^. Finally, one cross-sectional study in 1902 children (mean age 9.8 years) found no consistent association between myopia and disordered sleep, although there was an association between myopia and the “bedtime resistance” item at the Children’s Sleep Habits Questionnaire (CSHQ)^39^. To our knowledge, no study has investigated child sleep and hyperopia or the longitudinal association between sleep and refractive errors.

Based on the above literature review, we hypothesized that both shorter and longer sleep durations, and desynchronized circadian rhythm could be associated to refracting errors and subsequent eyeglasses prescription at a young age. We thus investigated, cross-sectionally and longitudinally, the associations between sleep duration and circadian timing (approximated by sleep timing with bedtime and midsleep)^40,41^, and refractive errors parentally declared among preschoolers from the EDEN birth-cohort.

## Methods

### Study population

The EDEN birth-cohort consists of 2002 pregnant women under 24 weeks of amenorrhea recruited between 2003 and 2006 in two French maternity hospitals (Poitiers and Nancy)^42^. It was designed in order to assess the pre- and postnatal determinants of child health and development. Women of less than 18 years-old, illiterate in French, with a history of diabetes and without social security coverage were not included in the cohort. Due to miscarriages, stillbirths and attrition, 1904 children were enlisted at birth.

Data of interest for this study were collected through parental self-filled questionnaires during pregnancy, and postnatally at 2 and 5 years.

In the present study, we excluded children whose parents never returned the 5 years questionnaire (n = 709), did not provide sleep information at both 2 and 5 years (n = 4), did not provide information regarding refraction disorders at 5 years (n = 31), declared that their child had a history of ophthalmic disease or non-hyperopic strabismus (n = 27) and diabetes (n = 1). Autistic children are known for their sleep disorders^43^ and sensory disorders^44^ and have therefore also been excluded (n = 1). Finally, it is also suspected that Black populations are more susceptible to visual disorders^6,45^. Racial statistics are prohibited in France, so we assimilated children whose maternal grandparents and paternal grandfather born in sub-Saharan Africa or in overseas France as being of black African descent, and we excluded them (n = 1). As a result, a total of 1130 children were included in our study.

Written informed consent was obtained twice from parents, once at enrolment and once after the child’s birth. The study was approved by the ethics research committee (Comité Consultatif de Protection des Personnes dans la Recherche Biomédicale) of the Bicêtre Hospital and by the Data Protection Authority (Commission Nationale de l’Informatique et des Libertés). All research was performed in accordance with relevant guidelines and regulations.

### Data collection

The existence of refractive errors was assessed on the 5 years old questionnaire, by the answers to the following 2 questions “Does your child have one of the following vision problems? Myopia: Yes/No/I don’t know; Hyperopia: Yes/No/I don’t know” and “Has he/she been prescribed eyeglasses? Yes/No” (eye glasses prescription is only made by vision specialist doctor in France). We defined three binary outcomes: eyeglass prescription for myopia, hyperopia or undeclared visual disorders (yes/no), existence of hyperopia (yes/no) and existence of myopia (yes/no).

Night sleep duration and midsleep were calculated from the daily bedtime and wake up time collected in the 2 and 5 year-questionnaires using the answers to the following questions at each age: “Usually, at what time does your child go to bed?”, “Usually, at what time does your child wake up?”. Responses were recorded in hours and minutes. We defined 6 exposures: nocturnal sleep duration, bedtime and midsleep (i.e. middle of night sleep), at age 2 and 5. Due to the physiopathology of eye growth and the conflicting results reported in the literature, we hypothesized that shorter and longer sleep durations would be a sensible proxy for maximal and minimal exposures to light/darkness, and would be associated with visual disorders in a non-linear manner, with both ends of the spectrum more susceptible to developing refraction errors. Therefore, we categorized the nocturnal sleep duration exposure variables. As we focused on nocturnal sleep duration, we could not use the thresholds recommended by the American Academy of Sleep Medicine (11–14 h per day for 1–2 year-old children and 10–13 h per day for 3–5 year-old children)^46^ that considered total sleep duration (i.e. including naps) and we decided to use tertile distribution with the middle one (AASM recommendation) as reference within the analyses. All sleep durations equal to the cut-off values were incorporated into the 2nd tertile as part of the medium sleep duration group, explaining why the tertile distributions, as shown in Table 1, do not display 33% in each group.

**Table 1:** Description of included children and comparison with excluded children for information collected at the maternity ward, before imputations.

	Included (n = 1130)		Excluded (n = 872)		
	% (n) or means (SD)	N Missing	% (n) or means (SD)	N Missing	*p* value
**Maternal characteristics**					
Maternal education (years)	14.0 (2.6)	4	12.9 (2.7)	88	< 10^−4^
Household income (€/month)		0		83	< 10^−4^
< 1500	11.4% (129)		25.2% (198)		
1500–3000	58.4% (658)		52.3% (411)		
> 3000	30.2% (340)		22.5% (177)		
**Child characteristics at birth**					
Sex (female)	47.4% (537)	0	47.5% (366)	99	0.95
Gestational age (weeks)	39.3 (1.7)	0	39.2 (1.8)	97	0.39
**Child characteristics at age 2**					
Nocturnal sleep duration (h/day)	11.1 (0.8)	187			
< 10h45	33.0% (311)				
10h45-11h30	44.4% (419)				
> 11h30	22.6% (213)				
Diurnal sleep duration (h/day)	2.1 (0.5)	118			
Bedtime (p.m.)	08.36 (30 min)	187			
Midsleep (a.m.)	02.06 (36 min)	187			
Age (years)	2.0 (0.1)	0			
**Child characteristics at age 5**					
Nocturnal sleep duration (h/day)	10.8 (0.5)	13			
< 10h30	15.3% (171)				
10h30-11h00	60.8% (681)				
> 11h00	23.9% (268)				
Bedtime (p.m.)	08.54 (30 min)	13			
Midsleep (a.m.)	02.06 (24 min)	13			
Daily outdoors time (hours)	1.6 (1.0)	59			
Daily screen time (hours)	1.4 (0.9)	35			
Age (years)	5.6 (0.2)	0			

Potential confounding factors were collected through questionnaires at different moments of the follow-up. Maternal education (in years of education) was collected in the pre-birth inclusion questionnaire. At birth were collected the sex of the child and the gestational age (in weeks of amenorrhea). In the 2 years questionnaire, household income was collected and categorized in “< 1500 €/month”, “1500–3000 €/month” and “> 3000 €/month”. In the 5-year questionnaire were collected the age and season at questionnaire completion, the daily amount of time spent watching screens (television, video games, computer) as well as the amount of time spent playing or walking outdoors, in hours per day.

### Statistical analyses

All analyses were performed using SAS software. Comparison of included to non-included participants were performed using Chi-squared test and Student’s t-tests.

Associations between sleep and refractive errors were analyzed separately for the three studied outcomes (i.e. eyeglass prescription, hyperopia, myopia) using logistic regressions. We performed cross-sectional analyses at age 5 and longitudinal analyses considering sleep duration and timing at age 2 and the existence of refractive errors at age 5. All analyses were first preadjusted for age at the 5 years questionnaire and maternity hospital. Multivariate logistic regressions were adjusted for confounding factors identified from the literature and selected using the Directed Acyclic Graphs (DAG) method (www.dagitty.net). All models were thus adjusted for maternity hospital, maternal age, maternal education, household income, child’s sex, gestational age, and time spent outdoors, daily screen time and season at the 5 years questionnaire completion. Longitudinal models including sleep duration were in addition adjusted for diurnal sleep at age 2. As bedtime and midsleep were strongly correlated with sleep duration (r = -0.32 and r = 0.40, respectively, both *p* < 10^–4^), these variables were not included in multivariate models.

Missing data (Table 1) were treated using multiple imputations based on all variables included in the adjusted models. This method assigned data to missing measurements based on the measurement of children with similar profiles. We assumed that data were missing at random and generated ten independent datasets with the fully conditional specification method (MI procedure, FCS statement, NIMPUTE option), and then calculated pooled effect estimates (MIANALYSE procedure) based on Rubin’s rules^47^.

## Results

### Sample description

Compared to non-included children, included children were more frequently from households with higher income (30% vs. 23% with income > 3000 €/month, *p* < 10^–4^) and more educated mothers (14 years vs. 13 years, *p* < 10^–4^) (Table 1).

The included children were 5.6 years old in average when questions on refraction were completed. They spent in average 1h36 (SD = 1 h) per day outdoors and 1h24 (54 min) in front of screens per day. Among the 1130 children included, 899 (79.6%) had no refractive errors reported, 231 (20.4%) had eyeglasses prescription, among whom 23 were myopic (2.0%) and 131 were hyperopic (11.6%). The reason for glasses prescription was unknown for 77 (6.8%). Mean nocturnal sleep duration was 11h05 (48 min) at age 2 and 10h49 (30 min) at age 5. Average diurnal sleep duration was 2h05 (30 min) at age 2. Tertile distribution of sleep durations at age 2 and 5 years are provided in Table 1. Average bedtime was 8.36 pm (30 min) at age 2 and 8.54 pm (30 min) at age 5. Midsleep was at 2.06 am on average (SD: 36 min at age 2, 24 min at age 5).

### Cross sectional and longitudinal analyses

Preadjusted and fully adjusted results are provided in Table 2. Cross sectional analyses at age 5 showed no significant association between sleep duration or timing and eyeglass prescription or existence of refraction troubles.

**Table 2:** Cross-sectional and longitudinal relations between each sleep characteristic separately (nocturnal sleep duration, bedtime and midsleep) and refractive errors at age 5 (n = 1130).

	Eyeglass prescription				Hyperopia				Myopia			
	OR (CI95%)*	*p* value	OR (CI95%)**	*p* value	OR (CI95%)*	*p* value	OR (CI95%)**	*p* value	OR (CI95%)*	*p* value	OR (CI95%)**	*p* value
**Cross-sectional analysis**												
Nocturnal sleep at age 5												
< 10h30	1.07 (0.70;1.62)	0.76	1.00 (0.65;1.53)	0.99	1.20 (0.74;1.93)	0.46	1.14 (0.69;1.87)	0.61	0.85 (0.28;2.60)	0.77	0.87 (0.28;2.72)	0.81
10h30-11h00	Ref		Ref		Ref		Ref		Ref		Ref	
> 11h00	1.25 (0.88;1.75)	0.21	1.28 (0.90;1.82)	0.17	1.21 (0.80;1.81)	0.36	1.23 (0.81;1.86)	0.33	0.59 (0.18;1.96)	0.39	0.58 (0.18;1.88)	0.36
Bedtime at age 5 (hours)	1.00 (0.72;1.40)	1.00	0.85 (0.61;1.20)	0.36	1.13 (0.77;1.64)	0.53	0.98 (0.65;1.49)	0.94	0.73 (0.29;1.79)	0.49	0.74 (0.30;1.88)	0.53
Midsleep at age 5 (hours)	1.09 (0.71;1.66)	0.69	0.94 (0.61;1.44)	0.76	1.27 (0.78;2.08)	0.34	1.11 (0.67;1.85)	0.68	0.76 (0.31;1.89)	0.56	0.77 (0.30;1.98)	0.58
**Longitudinal analysis**												
Nocturnal sleep at age 2												
< 10h45	1.43 (1.00;2.05)	0.05	1.42 (0.99;2.04)	0.06	1.25 (0.84;1.86)	0.27	1.25 (0.83;1.86)	0.28	1.27 (0.47;3.41)	0.63	1.29 (0.47;3.53)	0.62
10h45-11h30	ref		ref		ref		ref		ref		ref	
> 11h30	1.49 (1.01;2.22)	0.05	1.46 (0.97;2.19)	0.07	1.21 (0.77;1.91)	0.42	1.17 (0.73;1.86)	0.52	0.84 (0.24;2.94)	0.78	0.75 (0.20;2.85)	0.67
Bedtime at age 2 (hours)	1.33 (1.00;1.77)	0.05	1.20 (0.90;1.61)	0.22	1.37 (0.99;1.90)	0.06	1.26 (0.91;1.76)	0.17	1.00 (0.46;2.16)	1.00	1.07 (0.47;2.40)	0.87
Midsleep at age 2 (hours)	1.44 (1.10;1.89)	0.01	1.31 (0.99;1.74)	0.06	1.36 (0.98;1.88)	0.07	1.25 (0.90;1.72)	0.18	1.10 (0.57;2.12)	0.78	1.08 (0.52;2.25)	0.84

*Adjusted for maternity hospital recruitment and exact age at 5 years data collection.

**Additional adjustment for sex, gestational age, mother's length of schooling, household income, child's diurnal sleep at age 2, and season, daily time spent outdoors, and daily screen time at 5 years questionnaire completion.

**Figure 1:**
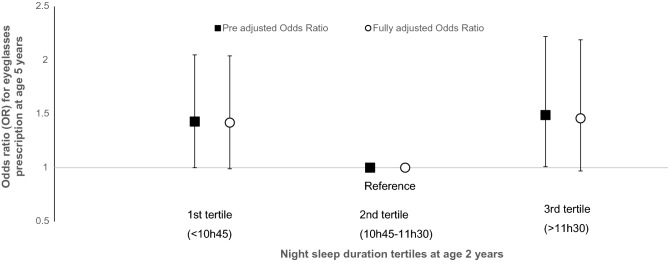
Odds ratios for eyeglasses prescription at age 5 according to sleep duration at age 2 (N = 1130).

However, in longitudinal analyses, when compared to children sleeping 10h45-11h30 at night at age 2 years, both children with night sleep duration < 10h45 and > 11h30 had a higher risk of eyeglasses prescription by the age of 5 (for myopia, hyperopia or unknown reasons), OR (95%CI) = 1.49 (1.01;2.22), *p* = 0.05 and OR = 1.43 (1.00;2.05), *p* = 0.05, respectively. Results were unchanged after adjustment for confounding factors although borderline significant (OR = 1.46 (0.97;2.19), *p* = 0.07 and OR = 1.42 (0.99;2.04), *p* = 0.06 when sleeping < 10h45 and when sleeping > 11h30, respectively) (Fig. 1).

Midsleep at age 2 was associated with an increased risk for eyeglass prescription (OR = 1.44 (1.10;1.89), *p* = 0.01). The association became borderline significant after adjustment (OR = 1.31 (0.99;1.74), *p* = 0.06). Bedtime was associated with an increased risk for eyeglasses prescription only in the raw analysis (OR = 1.33 (1.00;1.77), *p* = 0.05).

Midsleep and bedtime at age 2 were close to significance with hyperopia at age 5 (OR = 1.36 (0.98;1.88), *p* = 0.07 and OR = 1.37 (0.99;1.90), *p* = 0.06, respectively) but not anymore after adjustment. The other subgroup analyses considering separately each type of refraction error showed no significant association with sleep characteristics.

Daily screen time at age 5 was associated with higher risk of eyeglasses prescription at the same age (OR = 1.23 (1.04;1.44), *p* = 0.01), independently of the sleep characteristic studied, in all analyses. Results were unchanged after adjustment for confounding factors. No association was observed between daily time spent outdoors and refractive errors (Supplementary Tables).

## Discussion

In this first longitudinal study performed among preschoolers, we found a U-shaped association between sleep duration at age 2 and eyeglass prescription for refractive errors at age 5, and a linear positive association between midsleep at age 2 and eyeglass prescription at age 5. These results are in line with our hypotheses, based on animal and human studies literature review, that both shorter and longer sleep durations, and desynchronized circadian rhythm could be associated refracting errors and subsequent eyeglasses prescription at a young age. They provide further arguments for a possible causal effect of sleep (duration and timing) on eyesight maturation. Our results showed no significant association with myopia or hyperopia, but we were limited by the low proportion of cases within the studied cohort. Mild myopias might not have yet been detected because unnoticed before reading/writing activities in those preschoolers. On the contrary, hyperopia is often accompanied by headaches and eye convergence, which are more likely to be experienced as disabling before school age. This possible difference in refraction error detection and diagnosis could partly explain the large gap between our myopia and hyperopia prevalence. However, in subgroup analyses, although non-significant, the relation between sleep duration and hyperopia followed the same U-shape than for eyeglass prescription; while the relation was inversely linear with myopia, showing potential higher probability of myopia at age 5 for shorter sleeper at age 2.

While these longitudinal results need to be confirmed in larger cohorts, review of the literature showed that few publications focused on the relations between sleep and refractive errors in children. None focused on eyeglass prescription or hyperopia, making it difficult to compare our results. Only two recent longitudinal studies focused on sleep duration and myopia in children. The first one was performed in 1887 Chinese school-aged children (mean age 7 ± 0.4 years at baseline) and studied myopia progression over 4 years^48^. At baseline, children slept in mean 9.83 ± 0.58 h per night, their mean diopter measure was 0.98 ± 0.95. The mean myopia progression in 4 years was − 1.89 ± 1.28 diopters. The authors reported no significant association, in adjusted models, between baseline sleep duration as continuous variable or using tertile categorization (≤ 9.56/9.57–10.00/ ≥ 10.01 h per night) and myopia progression 4 years later. The second one, by Liu et al. also performed in Chinese school-aged children (6295 Chinese school-aged children (mean age 7.2 ± 0.7 years at baseline) reported no association in adjusted models between sleep duration considered in categories (< 9.5, 9.5–10, > 10 h per night) and myopia incidence and progression over 2 years, but a positive association between later bedtime (> 21h30) and myopia at baseline (6.8% of the children), 2-year myopia incidence (22%) and 2-year myopia progression^49^. However, we did not observe this association in our study. The discrepancies between studies may be explained by the differences in myopia measure and prevalence within each country, by the different children ages, myopia prevalence being lower in younger children and increasing with age, and by the confounding factors accounted for. However, while the association is non-significant in our study between shorter night sleep at age 2 (1st tertile, < 10h45) and myopia at age 5, probably due to lack of power, the odds ratio was positive. Follow up analyses in older children are needed to obtain results to be compared with the literature.

Three cross-sectional studies have reported a positive association between short sleep duration and myopia measured in diopter deficiency by a professional^7,37,38^. Two of these studies, reported associations between short sleep duration and myopia in adolescents. Jee et al*.* analyzed myopia in Korean adolescents (mean age 15.5) with a high myopia prevalence (77.8%) and short sleep (mean 7.1 h/night)^38^. They reported, in adjusted analyses, both an inverse linear association between sleep duration considered as a continuous variable and myopia (defined as at least -0.5 diopter) and lower odds ratio for adolescents to present myopia when sleeping > 8 h/night compared to those sleeping < 5 h. On the same line, Gong et al*.* showed in school-aged Chinese children (mean age 12.1 ± 3.3), with myopia defined as at least − 0.75 diopter (53.4% of the included children), a positive association between the two lower sleep durations tertiles (≤ 7 h and about 8 h/night) compared to the highest tertile (≥ 9 h/night) and myopia in adjusted models^7^. However, Ayaki et al*.* showed in Japanese adolescents from (mean age 14.1 ± 2.6) no association between sleep duration and mild (mean 2.56 ± 1.38 diopters) or high (mean − 7.5 ± 2.7 diopters) myopia after adjustment on confounding factors^37^. One study, also performed in Asia, in younger Chinese school-aged children (mean age 9.2 ± 0.44 years), showed that children with myopia (defined as at least − 0.5 diopter, 31%) slept 4 min longer than children without myopia and that night sleep duration was associated with higher odds for presenting myopia (1.02 (1.01;1.04), *p* = 0.002) after adjustment on confounding factors including sleep disorders measured by the Children Sleep Habit Questionnaire (CSHQ)^39^. This relation was no more significant when myopia was defined as at least -1 diopter, or when they considered total sleep duration per 24 h (i.e. adding naps). CSHQ subscale score analysis revealed that the bedtime resistance subscale was associated with myopia (defined as at least − 0.5 diopter, *p* = 0.005), hinting that myopia might be mainly attributed to sleep timing.

One of the studies used the PSQI to evaluate sleep durations in adolescents^37^, in which only nights < 7 h are considered of “bad quality”, while the recommendations for that adolescent age span are at least 8–9 h/night^46^. The same remark applies to Jee et al.’s study among adolescents where the mean sleep duration was 7.1 ± 0.0 h/night^38^. Gong et al. took the > 9 h category as reference, which is in line with the recommendations, but still had almost 10,000 individuals (62%) with shorter sleep durations^7^. These observations are coherent with the known cultural differences between European and Asian countries, which lead children to sleep significantly less in the latter^50^. On the contrary, in our included population, children had a mean total sleep duration of 13.2 ± 1.0 h/day (including naps) at age 2 while the consensus for children that age is 11–14 h/day, placing them in the higher recommendation ranges. In sum, former designs were different than ours (cross-sectional, myopia only) and the children included were older (mainly adolescents) and slept a lot less than the recommendations for their ages making a further comparison of our results in preschoolers unwise.

As described in the introduction and relying on former studies^12–15^, mainly in animals, the U-shaped relationship we observed, independently of time spent outdoors (a proxy of daylight exposure) and of screen time as we adjusted for, may be explained by the variation in nocturnal light exposure according to different sleep duration tertiles. Chicks have been shown to have axial length and corneal curvature modifications according to the amount of light or dark they were exposed to, turning them myopic or hyperopic, respectively. Retrospective case–control protocols have also been implemented in order to assess the importance of this phenomenon in children. An association was found between myopia and a history of nocturnal light exposure during the first 2 years of sleep (assessed by parental questionnaire) in 479 children between 2 and 16 years old^18^. Many studies with similar protocols and sample sizes varying from 122 to 1220 children did not replicate these findings^9,20,21,23,24^, but these studies were subject to memory bias. A larger case–control study (n = 3377) reported an association between myopia and a history of early nocturnal light exposure, although the analyses were not adjusted for confounders^22^. The evidence for a link between nocturnal light exposure and ametropias is hardly consistent, and whether our findings linking sleep duration to refractive errors are mediated by artificial light exposure is yet to be determined.

We also found an association between midsleep at age 2 and refractive errors at age 5. As said before, there is rising evidence linking biometric variations and diurnal rhythms which could have an impact on eye development and ultimately ametropias^28–30,32^. The results of our study suggest, accordingly to these previous studies, that a delay in the sleep phase could be a possible cause for the onset of refractive errors a couple of years later. A late midsleep could be the markers of a late endogenous chronotype, which is largely driven by the circadian timing system^51^. The delayed sleep could also be induced by a late exposure to light^52^, even for short durations^53^, relatively low intensity light emanating from LED screens^54^. It could also be the consequence of social schedules interfering with the biological time, known as social jetlag^55^. Social jetlag is mostly studied among adults with late chronotypes who suffer from school or work schedule, which is obviously irrelevant to our studied population, but it is likely that parental schedules interfere with their child’s chronotype, resulting there again in a disruption of circadian rhythmicity. Facing this unprecedented public health challenge, interventions to slow myopia progression in children are being studied, among which spectacles, contact lenses and pharmaceutical agents have a prominent place^56^. We advocate to also consider sleep and light hygiene for future interventions.

Our study has the advantage of being entirely prospective, reducing to a minimum the risk of recall bias in the assessment of the exposure variables. Our design allowed for a longitudinal analysis, while most of the pre-existing studies on the subject were cross-sectional, thus making a step towards the establishment of a possible causal relation between sleep and refraction. Moreover, we also included hyperopia in our design, hinting that there might be a relation between sleep and all refractive errors, and not just with myopia. There are however limitations as well. First, data were declarative by self-questionnaires. Sleep durations may be overestimated, especially since calculation was based on bedtime and wake time i.e. reflect more time in bed than objective sleep duration measured by actigraphy. However, actigraphy was not easily available at the time of the study and was not considered in the study design. We did not collect any information on the binocular vision or on the magnitude of the refraction error (ideally assessed with cycloplegic refraction during an eye examination). Some parents did not know why their child was prescribed eyeglasses, limiting our statistical power for the specific analyses on ametropias, especially myopia (n = 23). However, limiting the analysis to the children with known reasons for eyeglass prescription leads to the same results (despite a smaller sample size). We also lacked information on some adjustment factors. The heritability of refractive errors is high^35^, but the information on the existence of a parental refractive error was not collected, as well as near-work activity (reading/writing/close screens like pads and smartphones) that is a known risk factors of myopia onset^8^. Finally, the included children came from more educated and wealthier households than average in the EDEN cohort and on national scale. Further studies with larger sample size and clinical data on the intensity of the refraction error should be conducted.

## Conclusion

We have observed a U-shaped relation between sleep duration at age 2 and eyeglass prescription for refractive errors at age 5, and a positive linear association between midsleep at age 2 and eyeglass prescription at age 5. Subgroup analyses on myopia or hyperopia at age 5 showed no association with sleep but sample sizes were small. However, these results suggest the implication of sleep duration (particularly shorter and longer durations) and timing (delayed sleep phase) on the onset of subsequent refractive errors as early as in preschool age. Further investigations are therefore needed to confirm our results and assess the underlying mechanisms.

## Supplementary Information

Supplementary Information
