# Supplementary material for: Longitudinal association between sleep features and refractive errors in preschoolers from the EDEN birth-cohort
Source: Sci Rep. 2021 Apr 27;11:9044. doi: 10.1038/s41598-021-88756-w (PMC8079679; doi:10.1038/s41598-021-88756-w)
Supplement: Supplementary file 1 — Supplementary Information [file 41598_2021_88756_MOESM1_ESM.docx]

Supplementary Tables. Detailed preadjusted and fully adjusted models for cross-sectional and longitudinal relations between each sleep characteristic separately (nocturnal sleep duration, bedtime and midsleep) and refractive errors at age 5 (n = 1,130).

|  |  | **Eyeglass prescription** | | | | **Hyperopia** | | | | **Myopia** | | | |
| --- | --- | --- | --- | --- | --- | --- | --- | --- | --- | --- | --- | --- | --- |
|  |  | **OR (CI95%)*** | **p-value** | **OR (CI95%)**** | **p-value** | **OR (CI95%)*** | **p-value** | **OR (CI95%)**** | **p-value** | **OR (CI95%)*** | **p-value** | **OR (CI95%)**** | **p-value** |
| **Nocturnal sleep at age 5** | |  |  |  |  |  |  |  |  |  |  |  |  |
|  | < 10h30 | 1.07 (0.70;1.62) | 0.76 | 1.00 (0.65;1.53) | 0.99 | 1.20 (0.74;1.93) | 0.46 | 1.14 (0.69;1.87) | 0.61 | 0.85 (0.28;2.60) | 0.77 | 0.87 (0.28;2.72) | 0.81 |
|  | 10h30-11h00 | ref |  | ref |  | ref |  | ref |  | ref |  | ref |  |
|  | > 11h00 | 1.25 (0.88;1.75) | 0.21 | 1.28 (0.90;1.82) | 0.17 | 1.21 (0.80;1.81) | 0.36 | 1.23 (0.81;1.86) | 0.33 | 0.59 (0.18;1.96) | 0.39 | 0.58 (0.18;1.88) | 0.36 |
| Maternity hospital (Nancy) | | 0.81 (0.27;2.44) | 0.71 | 0.95 (0.70;1.30) | 0.77 | 0.94 (0.25;3.52) | 0.93 | 0.97 (0.67;1.40) | 0.87 | 0.77 (0.05;12.24) | 0.85 | 1.16 (0.47;2.86) | 0.74 |
| Age (years) |  | 0.91 (0.67;1.24) | 0.55 | 0.86 (0.28;2.60) | 0.79 | 0.93 (0.66;1.33) | 0.70 | 1.02 (0.27;3.85) | 0.98 | 1.26 (0.52;3.08) | 0.60 | 0.74 (0.04;12.5) | 0.83 |
| Sex (female) |  | 1.18 (0.88;1.57) | 0.27 | 1.19 (0.88;1.60) | 0.26 | 1.23 (0.88;1.72) | 0.24 | 1.24 (0.88;1.74) | 0.22 | 2.05 (0.92;4.57) | 0.08 | 2.10 (0.90;4.88) | 0.09 |
| Gestational age (weeks) | | 1.03 (0.94;1.13) | 0.53 | 1.03 (0.94;1.13) | 0.53 | 1.08 (0.96;1.21) | 0.19 | 1.08 (0.97;1.20) | 0.17 | 0.95 (0.74;1.23) | 0.70 | 0.94 (0.71;1.23) | 0.64 |
| Maternal education (years) | | 0.93 (0.88;0.99) | 0.02 | 0.94 (0.87;1.01) | 0.09 | 0.92 (0.85;1.01) | 0.07 | 0.92 (0.84;1.01) | 0.07 | 1.08 (0.89;1.30) | 0.44 | 1.12 (0.91;1.38) | 0.30 |
| Household income (€/month) | |  |  |  |  |  |  |  |  |  |  |  |  |
|  | < 1,500 | 1.49 (0.93;2.39) | 0.10 | 1.39 (0.85;2.26) | 0.19 | 1.37 (0.77;2.45) | 0.28 | 1.26 (0.71;2.23) | 0.44 | 1.53 (0.43;5.46) | 0.51 | 1.88 (0.48;7.27) | 0.36 |
|  | 1,500-3,000 | ref |  | ref |  | ref |  | ref |  | ref |  | ref |  |
|  | > 3,000 | 0.95 (0.69;1.30) | 0.75 | 1.18 (0.82;1.69) | 0.37 | 0.98 (0.66;1.46) | 0.94 | 1.28 (0.84;1.96) | 0.26 | 1.12 (0.48;2.59) | 0.79 | 0.94 (0.38;2.32) | 0.89 |
| Daily outdoors time at age 5 (hours) | | 0.95 (0.81;1.13) | 0.59 | 0.91 (0.76;1.10) | 0.33 | 0.98 (0.81;1.19) | 0.85 | 0.97 (0.79;1.19) | 0.79 | 0.98 (0.62;1.57) | 0.95 | 1.09 (0.64;1.86) | 0.76 |
| Daily screen time at age 5 (hours) | | 1.23 (1.04;1.44) | 0.01 | 1.21 (1.01;1.44) | 0.03 | 1.15 (0.89;1.49) | 0.27 | 1.10 (0.85;1.43) | 0.45 | 0.95 (0.56;1.59) | 0.83 | 1.02 (0.60;1.73) | 0.95 |
| Season at age 5 |  |  |  |  |  |  |  |  |  |  |  |  |  |
|  | Spring | ref |  | ref |  | ref |  | ref |  | ref |  | ref |  |
|  | Summer | 0.97 (0.62;1.51) | 0.88 | 0.97 (0.62;1.53) | 0.91 | 0.95 (0.56;1.62) | 0.86 | 0.97 (0.57;1.67) | 0.93 | 0.79 (0.23;2.70) | 0.70 | 0.74 (0.21;2.58) | 0.64 |
|  | Autumn | 0.89 (0.58;1.35) | 0.57 | 0.86 (0.56;1.32) | 0.49 | 0.94 (0.57;1.57) | 0.82 | 0.92 (0.55;1.55) | 0.77 | 0.82 (0.22;3.11) | 0.77 | 0.86 (0.22;3.31) | 0.82 |
|  | Winter | 1.13 (0.75;1.72) | 0.55 | 1.02 (0.66;1.58) | 0.93 | 1.28 (0.78;2.12) | 0.33 | 1.20 (0.73;1.99) | 0.47 | 1.04 (0.34;3.20) | 0.95 | 1.09 (0.33;3.64) | 0.89 |
| **Nocturnal sleep at age 2** | |  |  |  |  |  |  |  |  |  |  |  |  |
|  | < 10h45 | 1.43 (1.00;2.05) | 0.05 | 1.42 (0.99;2.04) | 0.06 | 1.25 (0.84;1.86) | 0.27 | 1.25 (0.83;1.86) | 0.28 | 1.27 (0.47;3.41) | 0.63 | 1.29 (0.47;3.53) | 0.62 |
|  | 10h45-11h30 | ref |  | ref |  | ref |  | ref |  | ref |  | ref |  |
|  | > 11h30 | 1.49 (1.01;2.22) | 0.05 | 1.46 (0.97;2.19) | 0.07 | 1.21 (0.77;1.91) | 0.42 | 1.17 (0.73;1.86) | 0.52 | 0.84 (0.24;2.94) | 0.78 | 0.75 (0.20;2.85) | 0.67 |
| Diurnal sleep at age 2 (by 15 min) | | 1.08 (1.01;1.16) | 0.03 | 1.09 (1.01;1.17) | 0.03 | 1.06 (0.96;1.15) | 0.24 | 1.06 (0.97;1.16) | 0.20 | 1.06 (0.88;1.28) | 0.51 | 1.09 (0.91;1.32) | 0.35 |
| Maternity hospital (Nancy) | | 0.81 (0.27;2.44) | 0.71 | 0.97 (0.71;1.33) | 0.85 | 0.93 (0.66;1.33) | 0.70 | 0.99 (0.68;1.43) | 0.94 | 0.77 (0.05;12.24) | 0.85 | 1.22 (0.49;3.02) | 0.67 |
| Age (years) |  | 0.91 (0.67;1.24) | 0.55 | 0.87 (0.28;2.67) | 0.80 | 1.23 (0.88;1.72) | 0.24 | 1.03 (0.27;4.00) | 0.96 | 1.26 (0.52;3.08) | 0.60 | 0.76 (0.04;13.9) | 0.85 |
| Sex (female) |  | 1.18 (0.88;1.57) | 0.27 | 1.19 (0.88;1.62) | 0.25 | 1.23 (0.88;1.72) | 0.24 | 1.25 (0.88;1.77) | 0.21 | 2.05 (0.92;4.57) | 0.08 | 2.25 (0.93;5.42) | 0.07 |
| Gestational age (weeks) | | 1.03 (0.94;1.13) | 0.53 | 1.03 (0.94;1.12) | 0.59 | 1.08 (0.96;1.21) | 0.19 | 1.07 (0.96;1.20) | 0.20 | 0.95 (0.74;1.23) | 0.70 | 0.94 (0.72;1.23) | 0.65 |
| Maternal education (years) | | 0.93 (0.88;0.99) | 0.02 | 0.94 (0.87;1.01) | 0.09 | 0.92 (0.85;1.01) | 0.07 | 0.92 (0.84;1.01) | 0.07 | 1.08 (0.89;1.30) | 0.44 | 1.12 (0.91;1.39) | 0.27 |
| Household income (€/month) | |  |  |  |  |  |  |  |  |  |  |  |  |
|  | < 1,500 | 1.49 (0.93;2.39) | 0.10 | 1.43 (0.87;2.36) | 0.16 | 1.37 (0.77;2.45) | 0.28 | 1.28 (0.72;2.29) | 0.40 | 1.53 (0.43;5.46) | 0.51 | 1.93 (0.50;7.35) | 0.34 |
|  | 1,500-3,000 | ref |  | ref |  | ref |  | ref |  | ref |  | ref |  |
|  | > 3,000 | 0.95 (0.69;1.30) | 0.75 | 1.17 (0.81;1.67) | 0.41 | 0.98 (0.66;1.46) | 0.94 | 1.26 (0.82;1.93) | 0.29 | 1.12 (0.48;2.59) | 0.79 | 0.92 (0.37;2.30) | 0.86 |
| Daily outdoors time at age 5 (hours) | | 0.95 (0.81;1.13) | 0.59 | 0.92 (0.76;1.10) | 0.35 | 0.98 (0.81;1.19) | 0.85 | 0.97 (0.80;1.19) | 0.80 | 0.98 (0.62;1.57) | 0.95 | 1.09 (0.64;1.83) | 0.76 |
| Daily screen time at age 5(hours) | | 1.23 (1.04;1.44) | 0.01 | 1.18 (0.99;1.41) | 0.07 | 1.15 (0.89;1.49) | 0.27 | 1.08 (0.84;1.40) | 0.52 | 0.95 (0.56;1.59) | 0.83 | 1.02 (0.59;1.76) | 0.95 |
| Season at age 5 |  |  |  |  |  |  |  |  |  |  |  |  |  |
|  | Spring | ref |  | ref |  | ref |  | ref |  | ref |  | ref |  |
|  | Summer | 0.97 (0.62;1.51) | 0.88 | 1.01 (0.64;1.59) | 0.97 | 0.95 (0.56;1.62) | 0.86 | 0.99 (0.57;1.69) | 0.96 | 0.79 (0.23;2.70) | 0.70 | 0.76 (0.21;2.67) | 0.67 |
|  | Autumn | 0.89 (0.58;1.35) | 0.57 | 0.89 (0.58;1.37) | 0.59 | 0.94 (0.57;1.57) | 0.82 | 0.94 (0.56;1.58) | 0.81 | 0.82 (0.22;3.11) | 0.77 | 0.86 (0.22;3.34) | 0.83 |
|  | Winter | 1.13 (0.75;1.72) | 0.55 | 1.08 (0.70;1.66) | 0.74 | 1.28 (0.78;2.12) | 0.33 | 1.24 (0.75;2.05) | 0.40 | 1.04 (0.34;3.20) | 0.95 | 1.10 (0.33;3.69) | 0.88 |
|  |  |  |  |  |  |  |  |  |  |  |  |  |  |
| ** adjusted on maternity hospital recruitment and exact age at 5 y data collection* | | | | |  |  |  |  |  |  |  |  |  |
| *** additional adjustment on sex, gestational age, mother's length of schooling, household income, child's diurnal sleep at age 2, and season, daily time spent outdoors, and daily screen time at 5 y questionnaire completion* | | | | | | | | | | | | | |
|  |  |  |  |  |  |  |  |  |  |  |  |  |  |
|  |  | **Eyeglass prescription** | | | | **Hyperopia** | | | | **Myopia** | | | |
|  |  | **OR (CI95%)*** | **p-value** | **OR (CI95%)**** | **p-value** | **OR (CI95%)*** | **p-value** | **OR (CI95%)**** | **p-value** | **OR (CI95%)*** | **p-value** | **OR (CI95%)**** | **p-value** |
| **Bedtime at age 5 (hours)** | | 1.00 (0.72;1.40) | 1.00 | 0.85 (0.61;1.20) | 0.36 | 1.13 (0.77;1.64) | 0.53 | 0.98 (0.65;1.49) | 0.94 | 0.73 (0.29;1.79) | 0.49 | 0.74 (0.3;1.88) | 0.53 |
| Maternity hospital (Nancy) | | 0.81 (0.27;2.44) | 0.71 | 0.93 (0.67;1.28) | 0.64 | 0.94 (0.25;3.52) | 0.93 | 0.98 (0.67;1.41) | 0.89 | 0.77 (0.05;12.24) | 0.85 | 1.10 (0.46;2.63) | 0.83 |
| Age (years) |  | 0.91 (0.67;1.24) | 0.55 | 0.83 (0.27;2.57) | 0.75 | 0.93 (0.66;1.33) | 0.70 | 0.97 (0.25;3.75) | 0.96 | 1.26 (0.52;3.08) | 0.60 | 0.77 (0.05;12.32) | 0.85 |
| Sex (female) |  | 1.18 (0.88;1.57) | 0.27 | 1.21 (0.90;1.64) | 0.20 | 1.23 (0.88;1.72) | 0.24 | 1.26 (0.90;1.78) | 0.18 | 2.05 (0.92;4.57) | 0.08 | 2.14 (0.91;5.03) | 0.08 |
| Gestational age (weeks) | | 1.03 (0.94;1.13) | 0.53 | 1.03 (0.94;1.12) | 0.59 | 1.08 (0.96;1.21) | 0.19 | 1.07 (0.96;1.19) | 0.21 | 0.95 (0.74;1.23) | 0.70 | 0.95 (0.73;1.23) | 0.69 |
| Maternal education (years) | | 0.93 (0.88;0.99) | 0.02 | 0.94 (0.87;1.01) | 0.09 | 0.92 (0.85;1.01) | 0.07 | 0.92 (0.84;1.01) | 0.87 | 1.08 (0.89;1.30) | 0.44 | 1.11 (0.91;1.37) | 0.30 |
| Household income (€/month) | |  |  |  |  |  |  |  |  |  |  |  |  |
|  | < 1,500 | 1.49 (0.93;2.39) | 0.10 | 1.38 (0.84;2.27) | 0.21 | 1.37 (0.77;2.45) | 0.28 | 1.22 (0.67;2.21) | 0.51 | 1.53 (0.43;5.46) | 0.51 | 1.86 (0.48;7.28) | 0.37 |
|  | 1,500-3,000 | ref |  | ref |  | ref |  | ref |  | ref |  | ref |  |
|  | > 3,000 | 0.95 (0.69;1.30) | 0.75 | 1.17 (0.81;1.68) | 0.40 | 0.98 (0.66;1.46) | 0.94 | 1.26 (0.82;1.93) | 0.29 | 1.12 (0.48;2.59) | 0.79 | 0.93 (0.37;2.33) | 0.87 |
| Daily outdoors time at age 5 (hours) | | 0.95 (0.81;1.13) | 0.59 | 0.91 (0.76;1.09) | 0.31 | 0.98 (0.81;1.19) | 0.85 | 0.97 (0.79;1.19) | 0.77 | 0.98 (0.62;1.57) | 0.95 | 1.08 (0.63;1.85) | 0.77 |
| Daily screen time at age 5 (hours) | | 1.23 (1.04;1.44) | 0.01 | 1.22 (1.02;1.46) | 0.03 | 1.15 (0.89;1.49) | 0.27 | 1.10 (0.85;1.43) | 0.47 | 0.95 (0.56;1.59) | 0.83 | 1.07 (0.63;1.83) | 0.80 |
| Season at age 5 |  |  |  |  |  |  |  |  |  |  |  |  |  |
|  | Spring | ref |  | ref |  | ref |  | ref |  | ref |  | ref |  |
|  | Summer | 0.97 (0.62;1.51) | 0.88 | 0.96 (0.61;1.51) | 0.86 | 0.95 (0.56;1.62) | 0.86 | 0.95 (0.55;1.63) | 0.86 | 0.79 (0.23;2.70) | 0.70 | 0.74 (0.21;2.57) | 0.63 |
|  | Autumn | 0.89 (0.58;1.35) | 0.57 | 0.84 (0.55;1.29) | 0.43 | 0.94 (0.57;1.57) | 0.82 | 0.9 (0.54;1.51) | 0.69 | 0.82 (0.22;3.11) | 0.77 | 0.86 (0.23;3.26) | 0.82 |
|  | Winter | 1.13 (0.75;1.72) | 0.55 | 0.98 (0.64;1.52) | 0.94 | 1.28 (0.78;2.12) | 0.33 | 1.16 (0.70;1.91) | 0.57 | 1.04 (0.34;3.20) | 0.95 | 1.06 (0.32;3.49) | 0.92 |
| **Bedtime at age 2 (hours)** | | 1.33 (1.00;1.77) | 0.05 | 1.20 (0.90;1.61) | 0.22 | 1.37 (0.99;1.90) | 0.06 | 1.26 (0.91;1.76) | 0.17 | 1.00 (0.46;2.16) | 1.00 | 1.07 (0.47;2.40) | 0.87 |
| Maternity hospital (Nancy) | | 0.81 (0.27;2.44) | 0.71 | 0.95 (0.68;1.33) | 0.77 | 0.93 (0.66;1.33) | 0.70 | 0.93 (0.63;1.38) | 0.72 | 0.77 (0.05;12.24) | 0.85 | 1.12 (0.46;2.76) | 0.80 |
| Age (years) |  | 0.91 (0.67;1.24) | 0.55 | 1.01 (0.32;3.20) | 0.99 | 1.23 (0.88;1.72) | 0.24 | 1.26 (0.33;4.86) | 0.74 | 1.26 (0.52;3.08) | 0.60 | 0.70 (0.04;13.00) | 0.81 |
| Sex (female) |  | 1.18 (0.88;1.57) | 0.27 | 1.20 (0.87;1.65) | 0.27 | 1.23 (0.88;1.72) | 0.24 | 1.24 (0.86;1.79) | 0.25 | 2.05 (0.92;4.57) | 0.08 | 1.83 (0.77;4.33) | 0.17 |
| Gestational age (weeks) | | 1.03 (0.94;1.13) | 0.53 | 1.03 (0.93;1.14) | 0.54 | 1.08 (0.96;1.21) | 0.19 | 1.07 (0.95;1.20) | 0.25 | 0.95 (0.74;1.23) | 0.70 | 0.98 (0.72;1.33) | 0.88 |
| Maternal education (years) | | 0.93 (0.88;0.99) | 0.02 | 0.93 (0.86;1.01) | 0.07 | 0.92 (0.85;1.01) | 0.07 | 0.92 (0.84;1.01) | 0.08 | 1.08 (0.89;1.30) | 0.44 | 1.13 (0.90;1.41) | 0.28 |
| Household income (€/month) | |  |  |  |  |  |  |  |  |  |  |  |  |
|  | < 1,500 | 1.49 (0.93;2.39) | 0.10 | 1.67 (0.98;2.87) | 0.06 | 1.37 (0.77;2.45) | 0.28 | 1.61 (0.87;2.98) | 0.13 | 1.53 (0.43;5.46) | 0.51 | 2.49 (0.65;9.59) | 0.18 |
|  | 1,500-3,000 | ref |  | ref |  | ref |  | ref |  | ref |  | ref |  |
|  | > 3,000 | 0.95 (0.69;1.30) | 0.75 | 1.27 (0.88;1.85) | 0.20 | 0.98 (0.66;1.46) | 0.94 | 1.41 (0.91;2.18) | 0.13 | 1.12 (0.48;2.59) | 0.79 | 0.87 (0.32;2.37) | 0.79 |
| Daily outdoors time at age 5 (hours) | | 0.95 (0.81;1.13) | 0.59 | 0.89 (0.74;1.09) | 0.27 | 0.98 (0.81;1.19) | 0.85 | 0.96 (0.77;1.19) | 0.70 | 0.98 (0.62;1.57) | 0.95 | 0.95 (0.52;1.73) | 0.87 |
| Daily screen time at age 5 (hours) | | 1.23 (1.04;1.44) | 0.01 | 1.17 (0.96;1.42) | 0.11 | 1.15 (0.89;1.49) | 0.27 | 1.08 (0.83;1.40) | 0.56 | 0.95 (0.56;1.59) | 0.83 | 0.95 (0.53;1.71) | 0.87 |
| Season at age 5 |  |  |  |  |  |  |  |  |  |  |  |  |  |
|  | Spring | ref |  | ref |  | ref |  | ref |  | ref |  | ref |  |
|  | Summer | 0.97 (0.62;1.51) | 0.88 | 1.03 (0.63;1.68) | 0.91 | 0.95 (0.56;1.62) | 0.86 | 1.08 (0.59;1.99) | 0.80 | 0.79 (0.23;2.70) | 0.70 | 0.60 (0.15;2.35) | 0.46 |
|  | Autumn | 0.89 (0.58;1.35) | 0.57 | 1.01 (0.63;1.61) | 0.96 | 0.94 (0.57;1.57) | 0.82 | 1.12 (0.63;2.02) | 0.69 | 0.82 (0.22;3.11) | 0.77 | 0.80 (0.20;3.16) | 0.75 |
|  | Winter | 1.13 (0.75;1.72) | 0.55 | 0.99 (0.61;1.61) | 0.98 | 1.28 (0.78;2.12) | 0.33 | 1.24 (0.70;2.19) | 0.45 | 1.04 (0.34;3.20) | 0.95 | 0.84 (0.24;2.88) | 0.78 |
|  |  |  |  |  |  |  |  |  |  |  |  |  |  |
| ** adjusted on maternity hospital recruitment and exact age at 5 y data collection* | | | | |  |  |  |  |  |  |  |  |  |
| *** additional adjustment on sex, gestational age, mother's length of schooling, household income, child's diurnal sleep at age 2, and season, daily time spent outdoors, and daily screen time at 5 y questionnaire completion* | | | | | | | | | | | | | |
|  |  |  |  |  |  |  |  |  |  |  |  |  |  |
|  |  | **Eyeglass prescription** | | | | **Hyperopia** | | | | **Myopia** | | | |
|  |  | **OR (CI95%)*** | **p-value** | **OR (CI95%)**** | **p-value** | **OR (CI95%)*** | **p-value** | **OR (CI95%)**** | **p-value** | **OR (CI95%)*** | **p-value** | **OR (CI95%)**** | **p-value** |
| **Midsleep at age 5 (hours)** | | 1.09 (0.71;1.66) | 0.69 | 0.94 (0.61;1.44) | 0.76 | 1.27 (0.78;2.08) | 0.34 | 1.11 (0.67;1.85) | 0.68 | 0.76 (0.31;1.89) | 0.56 | 0.77 (0.30;1.98) | 0.58 |
| Maternity hospital (Nancy) | | 0.81 (0.27;2.44) | 0.71 | 0.96 (0.70;1.33) | 0.82 | 0.94 (0.25;3.52) | 0.93 | 1.01 (0.70;1.48) | 0.94 | 0.77 (0.05;12.24) | 0.85 | 1.12 (0.47;2.64) | 0.80 |
| Age (years) |  | 0.91 (0.67;1.24) | 0.55 | 0.89 (0.29;2.73) | 0.84 | 0.93 (0.66;1.33) | 0.70 | 1.04 (0.28;3.93) | 0.95 | 1.26 (0.52;3.08) | 0.60 | 0.77 (0.05;12.22) | 0.85 |
| Sex (female) |  | 1.18 (0.88;1.57) | 0.27 | 1.17 (0.87;1.58) | 0.29 | 1.23 (0.88;1.72) | 0.24 | 1.24 (0.88;1.75) | 0.22 | 2.05 (0.92;4.57) | 0.08 | 2.13 (0.91;5.03) | 0.08 |
| Gestational age (weeks) | | 1.03 (0.94;1.13) | 0.53 | 1.02 (0.93;1.12) | 0.67 | 1.08 (0.96;1.21) | 0.19 | 1.07 (0.96;1.19) | 0.24 | 0.95 (0.74;1.23) | 0.70 | 0.95 (0.73;1.23) | 0.68 |
| Maternal education (years) | | 0.93 (0.88;0.99) | 0.02 | 0.94 (0.88;1.01) | 0.11 | 0.92 (0.85;1.01) | 0.07 | 0.92 (0.84;1.01) | 0.09 | 1.08 (0.89;1.30) | 0.44 | 1.12 (0.91;1.37) | 0.30 |
| Household income (€/month) | |  |  |  |  |  |  |  |  |  |  |  |  |
|  | < 1,500 | 1.49 (0.93;2.39) | 0.10 | 1.34 (0.81;2.21) | 0.26 | 1.37 (0.77;2.45) | 0.28 | 1.14 (0.62;2.11) | 0.67 | 1.53 (0.43;5.46) | 0.51 | 1.85 (0.47;7.36) | 0.38 |
|  | 1,500-3,000 | ref |  | ref |  | ref |  | ref |  | ref |  | ref |  |
|  | > 3,000 | 0.95 (0.69;1.30) | 0.75 | 1.16 (0.81;1.67) | 0.42 | 0.98 (0.66;1.46) | 0.94 | 1.26 (0.82;1.94) | 0.28 | 1.12 (0.48;2.59) | 0.79 | 0.94 (0.38;2.33) | 0.89 |
| Daily outdoors time at age 5 (hours) | | 0.95 (0.81;1.13) | 0.59 | 0.91 (0.76;1.09) | 0.32 | 0.98 (0.81;1.19) | 0.85 | 0.97 (0.79;1.18) | 0.75 | 0.98 (0.62;1.57) | 0.95 | 1.08 (0.64;1.85) | 0.76 |
| Daily screen time at age 5 (hours) | | 1.23 (1.04;1.44) | 0.01 | 1.20 (1.01;1.43) | 0.04 | 1.15 (0.89;1.49) | 0.27 | 1.09 (0.85;1.40) | 0.51 | 0.95 (0.56;1.59) | 0.83 | 1.05 (0.61;1.80) | 0.86 |
| Season at age 5 |  |  |  |  |  |  |  |  |  |  |  |  |  |
|  | Spring | ref |  | ref |  | ref |  | ref |  | ref |  | ref |  |
|  | Summer | 0.97 (0.62;1.51) | 0.88 | 0.94 (0.60;1.48) | 0.79 | 0.95 (0.56;1.62) | 0.86 | 0.95 (0.55;1.64) | 0.86 | 0.79 (0.23;2.70) | 0.70 | 0.75 (0.21;2.61) | 0.65 |
|  | Autumn | 0.89 (0.58;1.35) | 0.57 | 0.83 (0.54;1.28) | 0.39 | 0.94 (0.57;1.57) | 0.82 | 0.91 (0.54;1.53) | 0.71 | 0.82 (0.22;3.11) | 0.77 | 0.86 (0.22;3.34) | 0.83 |
|  | Winter | 1.13 (0.75;1.72) | 0.55 | 0.98 (0.63;1.52) | 0.93 | 1.28 (0.78;2.12) | 0.33 | 1.14 (0.69;1.89) | 0.61 | 1.04 (0.34;3.20) | 0.95 | 1.07 (0.32;3.56) | 0.92 |
| **Midsleep at age 2 (hours)** | | 1.44 (1.10;1.89) | 0.01 | 1.31 (0.99;1.74) | 0.06 | 1.36 (0.98;1.88) | 0.07 | 1.25 (0.90;1.72) | 0.18 | 1.10 (0.57;2.12) | 0.78 | 1.08 (0.52;2.25) | 0.84 |
| Maternity hospital (Nancy) | | 0.81 (0.27;2.44) | 0.71 | 1.02 (0.72;1.44) | 0.93 | 0.93 (0.66;1.33) | 0.70 | 0.96 (0.64;1.46) | 0.86 | 0.77 (0.05;12.24) | 0.85 | 1.28 (0.46;3.54) | 0.64 |
| Age (years) |  | 0.91 (0.67;1.24) | 0.55 | 1.16 (0.36;3.75) | 0.80 | 1.23 (0.88;1.72) | 0.24 | 1.38 (0.34;5.59) | 0.65 | 1.26 (0.52;3.08) | 0.60 | 0.74 (0.03;21.00) | 0.86 |
| Sex (female) |  | 1.18 (0.88;1.57) | 0.27 | 1.22 (0.87;1.71) | 0.24 | 1.23 (0.88;1.72) | 0.24 | 1.19 (0.80;1.77) | 0.39 | 2.05 (0.92;4.57) | 0.08 | 2.27 (0.87;5.93) | 0.09 |
| Gestational age (weeks) | | 1.03 (0.94;1.13) | 0.53 | 1.04 (0.94;1.15) | 0.44 | 1.08 (0.96;1.21) | 0.19 | 1.06 (0.94;1.20) | 0.32 | 0.95 (0.74;1.23) | 0.70 | 1.04 (0.71;1.51) | 0.85 |
| Maternal education (years) | | 0.93 (0.88;0.99) | 0.02 | 0.94 (0.87;1.02) | 0.16 | 0.92 (0.85;1.01) | 0.07 | 0.92 (0.84;1.02) | 0.10 | 1.08 (0.89;1.30) | 0.44 | 1.15 (0.90;1.48) | 0.27 |
| Household income (€/month) | |  |  |  |  |  |  |  |  |  |  |  |  |
|  | < 1,500 | 1.49 (0.93;2.39) | 0.10 | 1.95 (1.11;3.41) | 0.02 | 1.37 (0.77;2.45) | 0.28 | 1.87 (1.01;3.49) | 0.05 | 1.53 (0.43;5.46) | 0.51 | 3.02 (0.72;12.69) | 0.13 |
|  | 1,500-3,000 | ref |  | ref |  | ref |  | ref |  | ref |  | ref |  |
|  | > 3,000 | 0.95 (0.69;1.30) | 0.75 | 1.39 (0.94;2.06) | 0.10 | 0.98 (0.66;1.46) | 0.94 | 1.56 (0.98;2.49) | 0.06 | 1.12 (0.48;2.59) | 0.79 | 0.94 (0.33;2.69) | 0.90 |
| Daily outdoors time at age 5 (hours) | | 0.95 (0.81;1.13) | 0.59 | 0.91 (0.75;1.11) | 0.36 | 0.98 (0.81;1.19) | 0.85 | 0.95 (0.77;1.18) | 0.66 | 0.98 (0.62;1.57) | 0.95 | 1.07 (0.58;1.95) | 0.83 |
| Daily screen time at age 5 (hours) | | 1.23 (1.04;1.44) | 0.01 | 1.23 (1.00;1.51) | 0.05 | 1.15 (0.89;1.49) | 0.27 | 1.13 (0.86;1.49) | 0.38 | 0.95 (0.56;1.59) | 0.83 | 1.06 (0.60;1.87) | 0.84 |
| Season at age 5 |  |  |  |  |  |  |  |  |  |  |  |  |  |
|  | Spring | ref |  | ref |  | ref |  | ref |  | ref |  | ref |  |
|  | Summer | 0.97 (0.62;1.51) | 0.88 | 1.08 (0.64;1.82) | 0.76 | 0.95 (0.56;1.62) | 0.86 | 1.17 (0.62;2.19) | 0.63 | 0.79 (0.23;2.70) | 0.70 | 0.69 (0.16;2.93) | 0.62 |
|  | Autumn | 0.89 (0.58;1.35) | 0.57 | 1.14 (0.70;1.85) | 0.59 | 0.94 (0.57;1.57) | 0.82 | 1.26 (0.69;2.30) | 0.45 | 0.82 (0.22;3.11) | 0.77 | 0.96 (0.24;3.85) | 0.95 |
|  | Winter | 1.13 (0.75;1.72) | 0.55 | 0.97 (0.58;1.61) | 0.91 | 1.28 (0.78;2.12) | 0.33 | 1.24 (0.68;2.27) | 0.48 | 1.04 (0.34;3.20) | 0.95 | 0.82 (0.20;3.38) | 0.79 |
|  |  |  |  |  |  |  |  |  |  |  |  |  |  |
| ** adjusted on maternity hospital recruitment and exact age at 5 y data collection* | | | | |  |  |  |  |  |  |  |  |  |
| *** additional adjustment on sex, gestational age, mother's length of schooling, household income, child's diurnal sleep at age 2, and season, daily time spent outdoors, and daily screen time at 5 y questionnaire completion* | | | | | | | | | | | | | |
